# Exposure Assessment of Tropane Alkaloids via Barley Grain-Based Products Among Serbian Population

**DOI:** 10.3390/foods15030448

**Published:** 2026-01-27

**Authors:** Tijana Stojanović, Bojan Konstantinović, Vojislava Bursić, Gorica Vuković, Jelena Milešević, Milica Zeković, Ivana Šarac, Đorđe Pejin, Nataša Mandić, Milena Popov, Miroslav Agarski, Marijana Ćurčić

**Affiliations:** 1 Department of Environmental and Plant Protection, Faculty of Agriculture, University of Novi Sad, Trg Dositeja Obradovića 8, 21000 Novi Sad, Serbia; vojislava.bursic@polj.edu.rs (V.B.); natasa.mandic@polj.edu.rs (N.M.); milena.popov@polj.edu.rs (M.P.); agarskimiroslav@gmail.com (M.A.); 2 Field Test, Vinogradska 150b, 11000 Belgrade, Serbia; gorica.vukovic@fieldtest.rs; 3 Centre of Research Excellence in Nutrition and Metabolism, Institute for Medical Research, National Institute of Republic of Serbia, University of Belgrade, Tadeuša Košćuška 1, 11000 Belgrade, Serbia; jelena.milesevic@imi.bg.ac.rs (J.M.); milica.zekovic@imi.bg.ac.rs (M.Z.); ivana.sarac@imi.bg.ac.rs (I.Š.); 4 Department of Chemistry, Biochemistry and Environmental Protection, Faculty of Sciences, University of Novi Sad, Trg Dositeja Obradovića 3, 21000 Novi Sad, Serbia; djordje.pejin@dh.uns.ac.rs; 5 Department of Toxicology “Akademik Danilo Soldatović”, Faculty of Pharmacy, University of Belgrade, Vojvode Stepe 450, 11000 Belgrade, Serbia; marijana.curcic@pharmacy.bg.ac.rs

**Keywords:** atropine, scopolamine, risk assessment, organic production, conventional production, *Datura stramonium*, LC-MS/MS, human health

## Abstract

Dietary exposure to tropane alkaloids (TAs) in Serbia remains insufficiently investigated, while awareness among consumers and agricultural producers of potential exposure and related health risks, particularly for children, is low. Barley, a cereal widely used in food production, is still not included in the EU and Serbian regulations on maximum allowable atropine and scopolamine concentrations in food. However, the CONTAM panel established the group *ARfD* of 0.016 µg/kg bw/day for the sum of atropine and scopolamine. Therefore, a study was conducted on barley samples from organic and conventional production systems, in order to quantify the presence of atropine and scopolamine by LC-MS/MS. In all of the tested samples, both TAs were detected at concentrations above the LOD. The most contaminated sample was from the organic production, with the sum of atropine and scopolamine being 3.2 µg/kg. In order to evaluate the consumer risk from At and Sc in barley-based products, the EFSA framework for acute dietary exposure assessment was applied. The exposure was assessed for seven population groups consuming barley-based foods and beverages: toddlers, children, adolescents, adults, elderly, vegetarians, and pregnant women. The estimated daily intake, calculated according to the three consumption scenarios, did not exceed the established *ARfD* value. Since barley is not the only source of TA intake in Serbia, a prospective study on TA exposure should be performed in order to monitor TA concentrations, estimate exposure, and manage the risk.

## 1. Introduction

The United Nations’ (UN) 2030 Agenda emphasizes the importance of sustainable agriculture in ensuring food security while safeguarding natural resources and supporting climate neutrality goals. Over the past two decades, global agricultural production has increased substantially in response to the growing food demand, with total primary crop production reaching 9.9 billion t in 2023 [1,2]. Cereals consistently present the largest share of global crop production, yielding 3.1 billion t in 2023. The most commonly produced cereals worldwide are maize, rice, wheat, barley, and sorghum.

In terms of production, barley (*Hordeum vulgare* L.) ranks as the fourth most important cereal crop globally, with its cultivation dating back approximately 10,000 years [3]. Originating in the Fertile Crescent of the Middle East, its cultivation spread across Europe and the New World as agriculture and trade developed. Throughout history, it has been valued for promoting strength, health, and endurance, while many ancient cultures recognized its nutritional and medicinal properties [4].

Although modern barley production is largely driven by demand for livestock feed and the brewing industry, the interest in its use for human consumption has been increasing. This is mainly attributed to its high content of soluble dietary fiber, particularly *β*-glucan, which helps lower the glycemic index and cholesterol levels while supporting cardiovascular health. Consequently, barley is recognized as a functional grain, and barley-based products such as bread, biscuits, pasta, breakfast cereals, soups, and baby food are gaining importance on the market. Barley malt is also widely used in the food industry, particularly in flavorings and baked goods [4].

One of the major challenges affecting both agricultural production and food safety is weed infestation. Unlike the other pests, which occur periodically depending on various environmental and biological factors, weeds are a constant threat to crop production and are estimated to cause up to 34% yield losses globally [5].

In recent years, there has been a growing interest in plant-derived alkaloids, particularly tropane, pyrrolizidine, and opium alkaloids, due to their occurrence as contaminants originating from the weeds present in agricultural fields [6]. These alkaloids can contaminate the crops during the harvest and subsequently enter the food and feed products, potentially leading to adverse health effects following long-term exposure, while, in extreme cases, they may lead to death. Due to limited exposure data, the health risks associated with chronic dietary intake of these compounds are likely underestimated [6,7].

Tropane alkaloids (TAs) present a group of more than 300 secondary metabolites, which can be found in all parts of many plant species belonging to the Brassicaceae, Erythroxylaceae, Phyllanthaceae, Rhizophoraceae, Fabaceae, Moraceae, Olacaceae, Apocynaceae, Convolvulaceae, Proteaceae, and predominantly the family Solanaceae [8]. TAs are biosynthesized from ornithine or arginine via putrescine, resulting in the formation of the N-methyl-Δ^1^-pyrrolinium cation, which acts as the universal structural precursor of the tropane skeleton [9]. The bicyclic tropane ring is a defining element of their chemical structure [10]. TAs are predominantly esters of tropine (3*α*-tropanole), and, to a lesser extent, they occur as esters of pseudotropine (3*β*-tropanole). They can be broadly classified into three groups: TAs from the Solanaceae family (scopolamine and hyoscyamine), coca alkaloids from *Erythroxylum coca* (cocaine), and the recently identified group named calystegines (polyhydroxylated derivatives of nortropane, nortropane alkaloids—NTAs) [11]. Atropine and scopolamine present the most extensively studied TAs [12]. Another very extensively studied TA is hyoscyamine. Atropine (At) is a racemic mixture of (−)- and (+)-hyoscyamine, while only the (−)-enantiomer possesses the anticholinergic activity. Regarding scopolamine (Sc) and hyoscyamine, only their (−)-enantiomers are formed naturally, while (+)-enantiomers arise through racemization or synthetic processes [7].

The potential health impact of TAs present in food worldwide remains largely unclear, mainly due to the limited data on their occurrence. At and Sc act as anticholinergic compounds by inhibiting the binding of acetylcholine (neurotransmitter) to its muscarinic receptors. Their effects on the human peripheral nervous system (PNS) include dryness of the respiratory and gastrointestinal mucosa, mydriasis, and gastric and urinary retentions, as well as heart rate alterations. The central nervous system (CNS) is likewise affected, producing symptoms such as drowsiness, ataxia, hallucinations, difficulty concentrating, and impaired speech, as well as decreased blood pressure and heart rate. Owing to their toxicological relevance and recurrent detection in European food products, At and Sc are classified as undesirable substances in food and feed and have been regulated by the EU (European Union) since 2016 [13].

Human exposure to TAs most commonly occurs either through the consumption of contaminated food products or through intentional intoxication due to the misuse of TA-producing plants. Long-term dietary exposure to low TA concentrations may lead to chronic toxicity, while acute toxicity can result from ingestion of food with high TA levels [14]. In addition to contamination by the co-harvested TA-producing weeds, TAs may also persist in dust particles or even be transferred onto cereals through mechanical abrasion. Such a transfer may even occur within the harvesting equipment. Consequently, cereals can become contaminated, occasionally exceeding the maximum levels established by the EU. Also, the detection of TAs in a range of processed food products further suggests that they exhibit at least partial stability during common food-processing procedures [13]. Recent studies have even demonstrated the transfer of TAs from contaminated animal feed into products like cows’ milk, highlighting the pervasive nature of this risk throughout the food supply chain [15]. It is worth mentioning that the occurrence data for TAs in both feed (including roughage) and animal-derived foods are extremely scarce [13].

Recent literature also discussed the possibility of horizontal transfer of these alkaloids through the soil. The concept of HNPT (Horizontal Natural Product Transfer) refers to the release of secondary metabolites from donor plants (via plant residue decomposition or through root exudates) into the rhizosphere of the neighboring plants, by which they can be taken up through the root system [16,17]. TAs, such as At, can be absorbed by wheat and barley from the contaminated soil and then translocated into aerial tissues and grains [18,19]. The co-cultivation experiment revealed the uptake of the PAs by parsley, melissa, chamomile, peppermint, and nasturtium, which were grown adjacent to *Senecio jacobaea* [20]. Also, the pot trial study confirmed the accumulation of one of the PAs in rooibos plants when the residues of *S. burchellii* were applied [21]. These studies show that alkaloids can diffuse through the soil solution and be absorbed by the adjacent plants even without direct root contact. Furthermore, the field trials have indicated that PAs from PA-producing weeds can migrate into surrounding soil and surface waters, with an increase in their concentrations after the rainfall [22]. Such mobility within the agroecosystems suggests that TA contamination could occur not only via direct uptake by the crops, but also indirectly via non-producing weeds, which absorb TAs exuded from the donor plants (such as *Datura stramonium* L.). These secondary “acceptor” weeds could then act as additional vectors of contamination if co-harvested with the cereals, similarly to the primary TA-producing weeds. Although this indirect pathway has not yet been experimentally confirmed regarding the TAs, its plausibility is supported by the established evidence of the soil uptake of At in wheat, as well as by the aforementioned documented inter-plant transfer of alkaloids in other systems. A very recently published paper suggests that TAs and PAs may also occur in non-producing plants as a consequence of field cross-contamination and soil-mediated horizontal transfer, as well as pollination (bees previously visiting alkaloid-producing plants) [23].

In 2016, the EFSA (European Food Safety Authority) conducted a detailed study on TAs occurrence in plant-based foods across nine European countries, analyzing 1709 samples from June 2015 to August 2016 [24]. They tested various food categories, including cereals, vegetables, and herbal teas, for the presence of 24 TAs using the LC-MS/MS. The highest prevalence of TAs was noted in dried herbal teas, with 70.2% positive samples. The maximum concentration of TAs was recorded in a sample of dried herbal tea (4357.6 µg/kg). Cereal-based foods for children had the highest mean TA concentration of 130.7 µg/kg. The study concluded that At and Sc were the most frequently detected TAs and emphasized the need for ongoing monitoring in order to ensure food safety.

The health risk associated with the occurrence of TAs in food matrices has been further evidenced by recently reported cases of human poisoning resulting from the consumption of contaminated food products. In 2019, the food aid outbreak occurred in Uganda when people consumed the “Super Cereal” (cereals and soybean blend) contaminated with *D. stramonium* seeds. After ingestion, 315 people became sick, and 5 of them died [25]. In October 2022, several poisoning incidents occurred in Italy due to the consumption of spinach and spinach-based foods contaminated with TAs, with the leaves of *D. stramonium* being suspected as the source of the contamination [26]. A study reported this year presented a case of fatal poisoning in a 58-year-old male who ingested a meal contaminated with TAs, with toxicological analyses confirming the presence of At and Sc in all the analyzed postmortem biological samples [27]. Another alarming fact that unequivocally accentuates the problem of food contamination with TAs is that, in the last 5 years, over 50 notifications of hazards dealing with TAs were reported by the RASFF (Rapid Alert System for Food and Feed), with 48 of them being serious, while 4 were classified as potential risk/potentially serious [28].

In Serbia, the occurrence of TAs in food remains insufficiently investigated, while awareness among the agricultural producers regarding their potential health risks, particularly for children, is generally low. Moreover, barley is currently not included in either European Union or Serbian regulations concerning the maximum allowable levels of TAs, despite its widespread use in food products. Therefore, a two-year monitoring study was conducted on barley samples from the organic and conventional production systems. The study focused on the determination of At and Sc and the assessment of dietary exposure across different population groups, in order to compare the TA contamination risks between the production types.

The results of this research are intended to raise awareness among consumers and producers about the dangers associated with TAs and TA-producing weeds, as well as to encourage the introduction of systematic monitoring programmes and potential future expansion of the existing legislation. Although the other cereals also contribute to the overall exposure to TAs, this study focused on barley as an understudied cereal, while emphasizing that broader multi-commodity monitoring would enable direct comparison of the cereal-specific contributions.

## 2. Materials and Methods

### 2.1. Chemicals and Reagents

The reference standards of atropine and scopolamine were purchased from Sigma-Aldrich (St. Louis, MO, USA). The individual stock solutions were prepared in methanol at a concentration of 1 mg/mL. The working mixtures were then prepared by diluting the stock solutions with methanol to 10 and 1 µg/mL, and they were stored at −20 °C in the dark. Acetonitrile and methanol (HPLC Ultra Gradient grade) were obtained from J.T. Baker (Deventer, The Netherlands), while formic acid (analytical grade) was supplied by Fisher Scientific (Loughborough, UK). For extraction and clean-up, Hillium QuEChERS extraction pouches (550 mL, P/N QEHLL0510P) and dispersive kits (15 mL, P/N QDHLL15032) from Agilent Technologies (Santa Clara, CA, USA) were employed [29].

### 2.2. Instrumentation

An Agilent 1290 Infinity II HPLC (High-Performance Liquid Chromatography) system, equipped with a quaternary pump, a multisampler, and a thermostated column compartment, was employed for the determination of atropine and scopolamine. The chromatograph was coupled to an Agilent 6495 triple quadrupole mass spectrometer (LC/TQ), fitted with an Agilent Jet Stream Technology Ion Source (AJS ESI). The chromatographic separation was achieved on a Zorbax Eclipse Plus C18 Rapid Resolution HD Column (50 × 2.1 mm, 1.8 µm). The column temperature was maintained at 35 °C, with an injection volume of 2 µL. The mobile phase consisted of water (A) and methanol (B), both supplemented with 0.1% (*v*/*v*) formic acid, delivered in gradient mode at a flow rate of 0.25 mL/min. The gradient was programmed as follows: 5% B (1 min), linear increase to 40% B (7 min), further increase to 90% B (8 min) with a 2 min hold, then returned to the initial conditions within 1 min and re-equilibrated for 2 min. The total runtime was 11 min. ESI parameters were as follows: drying gas (nitrogen) at 200 °C and 16 L/min, nebulizer pressure at 30 psi, sheath gas of 300 °C at 12 L/min, and capillary voltage of 3000 V. The detection was carried out in Dynamic Multiple Reaction Monitoring (dMRM) mode. The method optimization and quantification were performed using Agilent MassHunter software (v.B.10.0 SR1, Agilent Technologies, 2006–2019, Santa Clara, CA, USA) [30].

### 2.3. Sample Collection and Preparation

Barley grain samples were collected from organic and conventional production systems across the representative agroecological regions of the Republic of Serbia during two consecutive growing seasons (2024 and 2025) at the following locations: Ljutovo, Njegoševo, Kikinda, Vajska, Kisač, Čurug, Rimski Šančevi, Martinci, Goričani, Kraljevo, Cerova, and Gnjilica.

The sampling was performed during the harvest by taking several spot subsamples directly from the trailer, which were subsequently combined into a composite sample weighing 1 kg. The composite samples were transferred to the University of Novi Sad, Faculty of Agriculture, Department of Environmental and Plant Protection. From each 1 kg field sample, an additional composite sample of 100 g was prepared, ground into powder, and stored in a refrigerator until analysis. From these 100 g samples, the subsamples of 5 ± 0.05 g were analyzed, while the remaining material was kept as a reserve. Sampling was performed following the principles of the Commission Implementing Regulation (EU) 2023/2783 [31]. The 5 g homogenized samples were placed into 50 mL PT cuvettes, mixed with 5 mL of water on a vortex for 20 s, and then left to soak. Afterwards, 20 mL of acetonitrile was added, followed by mixing on a shaker at 2500 rpm for 15 min. Then, the citrate salt mixture for the extraction was added, followed by mixing for 5 min on a shaker at 2500 rpm, after which they were centrifuged at 6000 rpm for 5 min. An aliquot of the supernatant (6 mL) was transferred into a cuvette containing adsorbents for the sample clean-up, mixed in a shaker at 2500 rpm for 1 min, and then centrifuged at 6000 rpm for 5 min. The obtained extract was filtered through a 0.45 µm microfilter and analyzed by the LC-MS/MS (Liquid Chromatography–Tandem Mass Spectrometry).

### 2.4. Nutrient Composition of the Barley Grain Samples

The nutrient composition of the barley grain samples collected from organic and conventional production systems was analyzed using a DA 7250™ Diode Array Near-Infrared Analysis System (PerkinElmer, Inc., Waltham, MA, USA). The analysis was carried out using a rapid and accurate indirect method, the precision of which depends on the accuracy of the reference methods used for the calibration of the instrument. A representative surface area of the sample was scanned, without the need for additional sample preparation. The instrument operated in down-view reflection or transflectance mode. The detector wavelength range was 900–1700 nm, with an operational range of 950–1650 nm and a resolution of <0.05 nm. The detector was a thermoelectrically cooled, 256-pixel indium gallium arsenide (InGaAs) array. A sufficient amount of each of the samples was placed into small sample trays to fully cover the measurement window, and the surface was leveled using a ruler with gentle shaking. Each sample was analyzed in five replicates using both rotating and static tray modes. The following traits were measured without the additional processing of the samples: moisture, protein, dry and wet gluten, hardness, starch, W (alveograph energy value), Zeleny sedimentation value, and NDF (neutral detergent fiber). The predefined minimum and maximum operational ranges were set as follows: moisture 7.3–22.1%, protein 8.2–22.9%, dry gluten 4.9–13.1%, wet gluten 16.7–39.6%, hardness 12.9–87.2, starch 61.5–83%, W 41–450, and Zeleny 16–54. NDF values were obtained using the available NDF calibration model and are reported on a dry matter basis.

### 2.5. Validation Parameters

At and Sc were quantified using electrospray ionization in positive mode (ESI+), applying dMRM. The fragmentation of the protonated At and Sc generated three product ions for each compound. For quantification, the most abundant MRM transitions were monitored (*m*/*z* 290.20 → 124.20 for atropine; *m*/*z* 304.20 → 156.00 for scopolamine), while for confirmation, the less intense transitions were used (Table 1).

**Figure 1:**
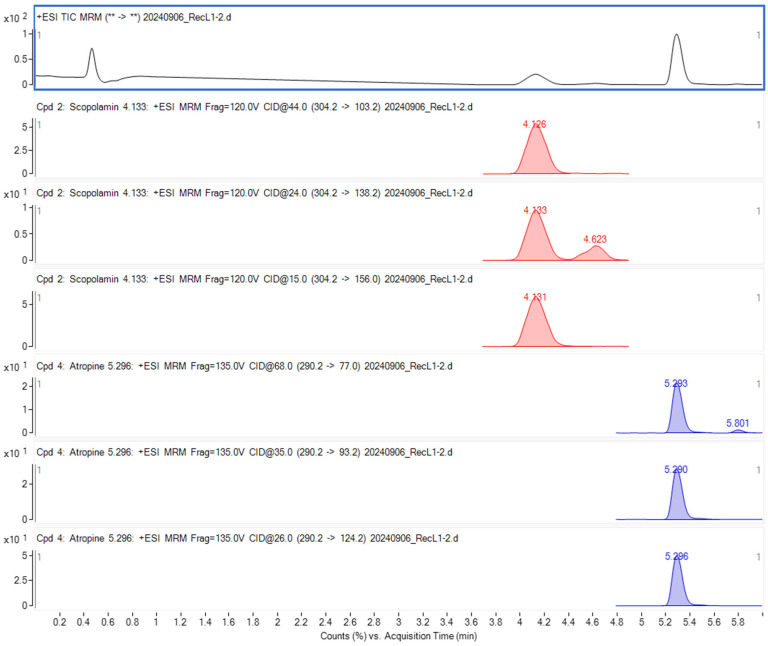
At and Sc chromatograms obtained by the LC-MS/MS.

The chromatographic conditions were the same as those described in a previous study [32]. The MRM chromatograms, as well as the mass spectra corresponding to the monitored transitions of the investigated TAs, are presented in Figure 1.

The retention times (Rt) for At and Sc were 5.30 and 4.10 min, respectively. The LOD (Limit of Detection), determined from the signal-to-noise ratio calculated using MassHunter software, was 0.5 µg/kg. The LOQ (Limit of Quantification) was established at 2 µg/kg for both At and Sc across the matrices, in accordance with the LOQ requirements outlined in [31] and SANTE 11312/2021 [33].

The standard solutions of TAs were spiked at five calibration levels: 1, 2, 5, 10, and 20 µg/kg. The concentrations of At and Sc in the samples were determined using the corresponding calibration curves for each compound. The correlation coefficients (R^2^) together with the mean recoveries at 2 and 10 µg/kg (Rec, %) and repeatability (%RSDr), as well as the expanded uncertainty (Up, %), are presented in Table 2.

A pronounced matrix effect (ME, %) was observed for both At and Sc in the barley matrix, as evaluated by comparison of the slopes of solvent-based and matrix-matched calibration curves. The ME was −92.6% for At and −90.3% for Sc, indicating very strong signal suppression for both analytes. Such values are unacceptable for reliable quantification using the solvent-based calibration. Therefore, the quantification in barley grain must be performed using matrix-matched calibration in order to compensate for matrix-induced signal suppression and to ensure the accuracy and reliability of the analytical results, in accordance with [33,34].

### 2.6. Barley-Based Food and Beverage Intake and Consumers’ Anthropometric Data

The data on barley-based food and beverage intake were obtained from the Serbian National Food Consumption Survey (SNFCS) for seven population groups (toddlers, children, adolescents, adults, elderly, vegetarians, and pregnant women), which was designed and carried out in accordance with the principles, methodological framework, and guidelines of the EU Menu project during the 2017–2021 period. Five population groups were defined according to sex and age (years, y), comprising male and female: toddlers (1 to <3 y), children (3–9 y), adolescents (10–17 y), adults (18–64 y), and elderly (65–74 y). In addition to these groups, two ad hoc categories (vegetarians and pregnant women) were included independently of age or sex [35,36].

### 2.7. Exposure Assessment

Consumption amounts were taken directly from the SNFCS dietary records. The exposure scenarios were constructed to estimate the proportion of barley in composite foods where quantitative ingredient information was unavailable, and not to model the consumption patterns. The exposure and risk assessment were performed in accordance with the EFSA guidance for acute dietary risk assessment of TAs [37]. In accordance with the EFSA human health risk assessment framework, the present evaluation follows a four-step approach consisting of (1) hazard identification, (2) dose–response (hazard characterization), (3) exposure assessment, and (4) risk characterization. The hazard identification and dose–response modelling for At and Sc (including acute toxicity and critical effect levels) have been previously established by the EFSA [38] based on extensive in vivo and clinical toxicological data and are, therefore, taken as a starting point for the present study. These evaluations provide the health-based guidance value (*ARfD*) used in this research. The present work focuses on phases (3) and (4), in which the dietary exposure to At and Sc from the barley-based products is estimated and compared with the EFSA reference value in order to characterize the consumer risk. Based on the SNFCS data, the consumers of barley-based foods and beverages were identified within the seven population groups. For each group, the estimated daily intake (*EDI*) was calculated for three consumption scenarios for both organic and conventional production, using consumer body weight, the recorded consumption of the barley-based foods and beverages for each 24-h dietary recall period across the two non-consecutive diary replicates, as well as the At and Sc concentration data obtained by the LC-MS/MS. For each production system, the highest detected At concentration, the highest detected Sc concentration, as well as the highest sample-specific calculated sum of At and Sc were used as the concentration inputs across the three consumption scenarios, according to Equation (1):

*EDI* = (*c* × *C*)/*bw* (1)

where *c* is the highest recorded concentration of At or Sc, or the sum of At and Sc by the LC-MS/MS (µg/kg), *C* is the total daily amount of the consumption of the barley-based foods and beverages (kg), and *bw* is the body weight of the consumer (kg).

Subsequently, for all calculated *EDI* values (for organic and conventional production, for At, Sc, and the sum of At and Sc, and for all three consumption scenarios), the maximum, median, and average values were determined, as well as the 75th (P75) and 95th (P95) percentiles. In order to improve the clarity and comparability of the results, all values were converted to nanograms (ng) by dividing them by 1000. Finally, the Hazard Quotient (*HQ*) was calculated for the mentioned values according to Equation (2):

*HQ* = *EDI*/*ARfD* (2)

where *EDI* is the estimated daily intake for At or Sc, or the sum of At and Sc for one of the three consumption scenarios for organic or conventional production, while *ARfD* is the value of 16 ng/kg bw/day for the group acute reference dose, established by the EFSA [38].

The calculations were performed in Excel (v. 2013, Microsoft, 2013–2023, Washington, DC, USA), separately for both conventional and organic production, as well as for the three different consumption scenarios: the realistic scenario (lower realistic scenario), the conservative scenario (upper realistic scenario), and the extremely conservative scenario (maximum worst-case scenario).

The assessment of barley intake was conducted by identifying all foods and beverages in the dietary records that could contain barley or barley-derived ingredients. For each reported meal, the potential presence of barley was evaluated based on food composition data (provided recipes), typical industrial formulations, and publicly available product labels, as well as the known technological practices relevant to the Serbian food market. Since quantitative declarations for barley-containing ingredients were generally unavailable, three exposure scenarios were constructed to estimate the possible amount of barley per 100 g of the consumed product.

The realistic scenario (Realistic-Case Scenario, RCS) presents the most probable barley content in each food and beverage. It was derived by considering the typical proportion of the barley-containing ingredient in comparable commercial products or standard recipes, the technological role of barley or barley derivatives in that type of food, and the expected contribution of that ingredient to the final mass of the product. When barley derivatives, such as barley malt or barley malt syrup, were present only in minor technological amounts, very low values were assigned. This scenario, therefore, reflects the best estimate of true exposure under normal consumption conditions.

The conservative scenario (Worst-Case Scenario, WCS) presents a deliberately higher, yet still technologically plausible, estimate of the barley content. In this case, the upper bound of the realistic range was assigned to each food and beverage, with the assumption that the barley-containing ingredient might be present in somewhat larger proportions than typically expected. This scenario does not represent an extreme value, but provides an intentionally cautious estimate suitable for protective exposure assessment.

The maximum (worst-case) scenario (Extremely Worst-Case Scenario, EWCS) reflects the highest technologically feasible amount of barley that could be present in a given product. The scenario assumes that the maximum reasonable portion of the formulation could originate from the barley-containing ingredient. This scenario does not describe realistic exposure, but defines the theoretical upper limit necessary for bounding the risk.

For each food item, the barley content calculated under a given scenario was proportionally adjusted to the reported portion size. The daily intake was obtained by summing the scenario-specific barley contributions for each of the consumers from all the foods and beverages consumed over the assessed day. This approach allowed the estimation of the typical, cautious, and upper-bound dietary exposure to barley for all the studied participants across the seven population groups.

### 2.8. Statistical Analysis

The obtained results were analyzed by one-way and factorial ANOVA (analysis of variance), as well as Tukey’s HSD (Honestly Significant Difference) post hoc test in Statistica (v. 14.1.08.; TIBCO Software Inc., Palo Alto, CA, USA, University Licence). The same letters next to the values denote the same level of statistical significance (*p* < 0.05).

The correlation between the nutrient composition of the barley samples and the sum of At and Sc was evaluated using the Spearman’s rank correlation coefficient, as a non-parametric method, as well as the Pearson’s correlation coefficient as a complementary parametric approach to compare the overall correlation trends.

## 3. Results

### 3.1. Tropane Alkaloids in Barley Grain Samples from Organic and Conventional Production

In all of the tested samples, both TAs were detected at concentrations above the LOD. The concentrations of At above 1 µg/kg were observed in 60% of organic and 14.29% of conventional barley samples. The concentrations of Sc above 1 µg/kg were noted for 40% of organic and 14.29% of conventional barley samples. The maximum and minimum values of At, Sc, and the sum of At and Sc observed in the organic and conventional barley samples in this study are shown in Table 3.

### 3.2. Estimated Daily Intakes

Table 4 presents the calculated *EDI* values for the sum of At and Sc under three exposure scenarios and across seven population groups. The values are based on the highest concentrations of the sum of At and Sc detected in barley samples from organic and conventional production.

### 3.3. Statistical Analysis

**Figure 2:**
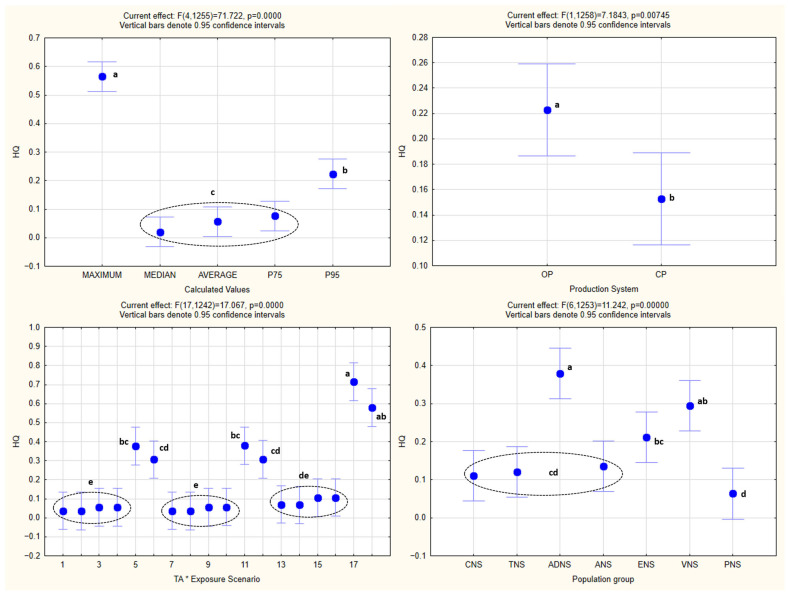
One-way analysis of the obtained *HQ* values. The same letters next to the values denote the same level of statistical significance (*p* < 0.05). The dashed circles and brackets indicate closely positioned values sharing the same level of statistical significance. TNS—toddlers (1 to <3 y); CNS—children (3–9 y); ADNS—adolescents (10–17 y); ANS—adults (18–64 y); ENS—elderly (65–74 y); VNS—vegetarians; PNS—pregnant women; OP—Organic production; CP—Conventional production; Exposure scenarios (first 9 for organic and second 9 for conventional production): At RCS 1st (Atropine, Realistic-Case Scenario, 1st day); At RCS 2nd (Atropine, Realistic-Case Scenario, 2nd day); At WCS 1st (Atropine, Worst-Case Scenario, 1st day); At WCS 2nd (Atropine, Worst-Case Scenario, 2nd day); At EWCS 1st (Atropine, Extremely Worst-Case Scenario, 1st day); At EWCS 2nd (Atropine, Extremely Worst-Case Scenario, 2nd day); Sc RCS 1st (Scopolamine, Realistic-Case Scenario, 1st day); Sc RCS 2nd (Scopolamine, Realistic-Case Scenario, 2nd day); Sc WCS 1st (Scopolamine, Worst-Case Scenario, 1st day); Sc WCS 2nd (Scopolamine, Worst-Case Scenario, 2nd day); Sc EWCS 1st (Scopolamine, Extremely Worst-Case Scenario, 1st day); Sc EWCS 2nd (Scopolamine, Extremely Worst-Case Scenario, 2nd day); At+Sc RCS 1st (Sum of Atropine and Scopolamine, Realistic-Case Scenario, 1st day); At+Sc RCS 2nd (Sum of Atropine and Scopolamine, Realistic-Case Scenario, 2nd day); At+Sc WCS 1st (Sum of Atropine and Scopolamine, Worst-Case Scenario, 1st day); At+Sc WCS 2nd (Sum of Atropine and Scopolamine, Worst-Case Scenario, 2nd day); At+Sc EWCS 1st (Sum of Atropine and Scopolamine, Extremely Worst-Case Scenario, 1st day); At+Sc EWCS 2nd (Sum of Atropine and Scopolamine, Extremely Worst-Case Scenario, 2nd day); P75—75th percentile; P95—95th percentile.

**Figure 3:**
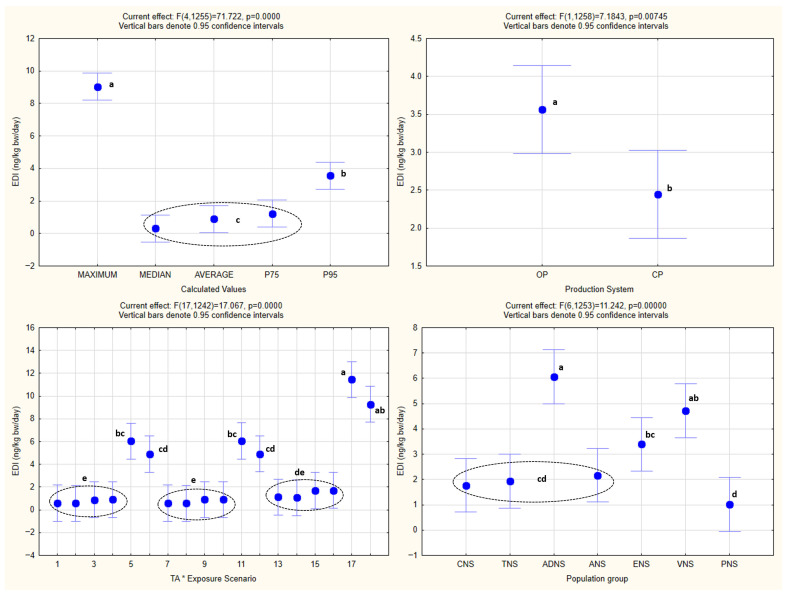
One-way ANOVA of *EDI* values. All abbreviations, symbols, and statistical notations follow the definitions provided in Figure 2.

**Figure 4:**
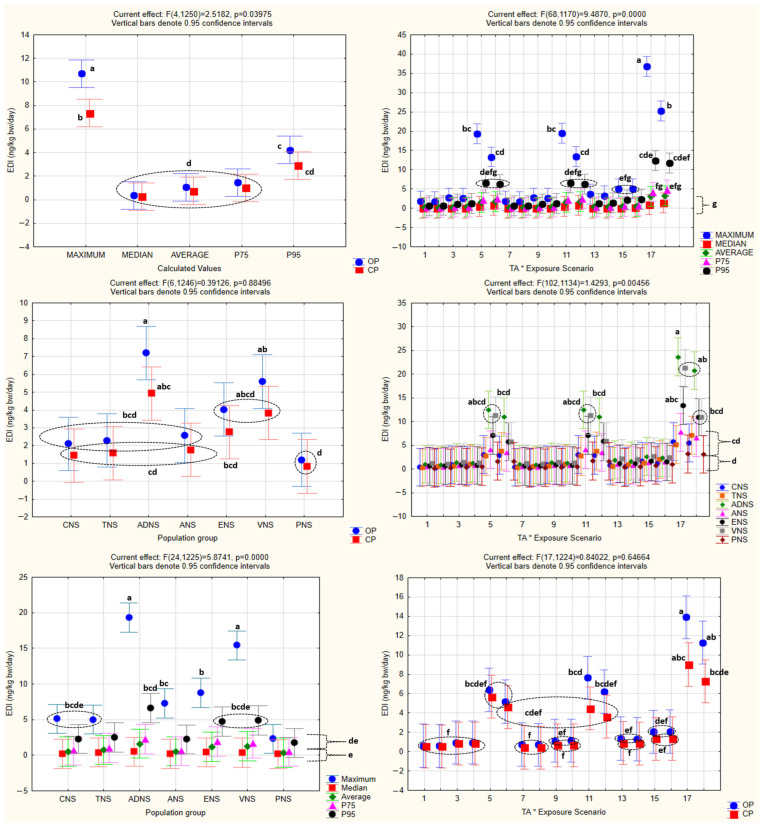
Factorial ANOVA of *EDI* values. All abbreviations, symbols, and statistical notations follow the definitions provided in Figure 2.

The analysis of the obtained *HQ* values by one-way ANOVA is shown in Figure 2, while one-way and factorial ANOVA analyses of *EDI* values, with the statistically significant homogenous groups obtained by Tukey’s HSD post hoc test, are presented in Figure 3 and Figure 4.

One-way ANOVA confirmed the existence of statistical significance for the effect of every one of the independent variables (population groups, production systems, exposure scenarios, and calculated values) (*p* < 0.05) on *EDI* as the dependent variable.

The same was noted in the case of factorial ANOVA for every one of the combined effects of the independent variables, with the exception of the combined effect of the population groups and production systems (*p* = 0.88), as well as the exposure scenarios and production systems (*p* = 0.65).

### 3.4. Nutrient Compositions of the Barley Grain Samples and Their Correlation with the TA Content

Based on the mean values of five replicate measurements for each sample, the analyzed grain quality parameters ranged as follows: moisture from 8.22 to 11.00%, protein (dry basis) from 8.17 to 12.36%, dry gluten from 7.12 to 14.01%, wet gluten from 19.54 to 25.94%, hardness from 45.89 to 70.51, starch (dry basis) from 44.84 to 67.56%, W from −94.80 to 57.61, Zeleny sedimentation value from −77.97 to 10.33 mL, and NDF from 23.84 to 37.59% (dry basis).

Spearman’s correlation analysis revealed a statistically significant positive association between the sum of At and Sc and the protein content (r_s_ = 0.76, *p* < 0.05). In addition, positive trends were observed for dry gluten content (r_s_ = 0.62), while a negative trend was found for starch content (r_s_ = −0.48). However, these associations did not reach statistical significance (Table 5). Pearson’s correlation analysis demonstrated the same directional relationships, with generally higher correlation coefficients, confirming the consistency of the observed trends across both analytical approaches.

## 4. Discussion

### 4.1. Tropane Alkaloids in Barley Grain Samples from Organic and Conventional Production

The obtained results showed that At and Sc occurred in all of the analyzed barley samples from organic and conventional production at generally low concentrations. However, notable differences were observed between the production systems. Organically produced barley exhibited not only higher maximum concentrations, but also a greater percentage of the samples exceeding 1 µg/kg compared to the conventionally produced barley. This pattern is plausibly linked to weed-management practices, as organic production does not allow the use of synthetic herbicides, making the weed control of the TAs producing weed species more challenging.

To date, only very limited monitoring data are available on TAs in barley grain. The published occurrence studies and reviews have mostly focused on other cereal matrices (e.g., millet, sorghum, buckwheat, and multigrain cereal-based baby foods), while barley is either under-represented or not reported separately in the datasets [12]. In the FAO/WHO compilation of the monitoring data, only a few barley grain samples were included, while none of them showed detectable levels of At and Sc [39]. Consequently, the direct comparison of the results in this study with barley-specific data from other countries is not feasible, and the present research provides one of the first systematic datasets on At and Sc in barley produced in Serbia.

### 4.2. Nutrient Composition of the Barley Grain Samples and Its Correlation with the TAs Content

The observed associations between the sum of At and Sc and the analyzed barley grain quality parameters suggest that the contamination is not uniformly distributed across the grain, but is linked to the specific grain fractions. Higher TAs levels tended to occur in the samples richer in protein and gluten and relatively poorer in starch. This indicates that TAs are associated with the non-endosperm components of the grain, such as the outer layers and accompanying plant material, rather than with the starch-rich endosperm itself. Since TAs are produced by the weeds, and not by barley, their presence in the grains is most plausibly explained by the co-harvesting of the weed seeds, plant fragments, or dust. Consequently, the technological steps that remove the outer grain layers or foreign plant material, such as cleaning, sorting, and dehulling, can be expected to reduce the TA levels in barley-based products.

### 4.3. Estimated Daily Intakes

The initial exposure assessment based on EWCS in some cases produced *HQ* values greater than 1 for the sum of At and Sc. EWCS assumed the maximum technologically feasible contribution of barley to a product, and as such, it generally presents the baseline framework of any exposure assessment prior to the application of the refined and more realistic scenarios. Following this preliminary step, the exposure was recalculated using realistic and conservative scenarios, which more accurately reflect the actual food composition and consumption patterns. Under both refined scenarios (RCS and EWCS), all the *HQ* values decreased and remained below 1, indicating that the exceedances observed under EWCS do not translate into a plausible health risk under real dietary conditions. The estimated daily intake in RCS and EWCS did not exceed the established *ARfD* value of 16 ng/kg bw/day.

These findings are comparable to the previously published exposure assessment studies for other cereal matrices. In Korea, similarly low dietary exposure across all the age groups to At and Sc in millet samples was reported, with the mean value of 0.23 ng/kg bw/day and no indication of acute health concern even under extreme consumption scenarios [40]. In a combined dataset of over 280 samples, the majority of flours, maize products, bread, pasta, and breakfast cereals showed minimal contamination, while higher levels were mainly associated with millet and sorghum flours, including one millet sample with a total TA content of 33.6 µg/kg. Cereal-based infant foods exceeded 1 µg/kg in 4 out of 66 UK samples [41]. A recent study on multigrain cereal-based baby products detected At and Sc in all the analyzed samples, although concentrations remained low, ranging from 0.07 to 1 µg/kg for At and from 0.15 to 12 µg/kg for scopolamine [42]. The EFSA’s evaluation, based on more than 44,000 analytical results from 17 European countries, indicated that the highest mean and 95th percentile exposures to the sum of At and Sc occur in infants, toddlers, and young children, with the estimated mean intakes ranging from 1 to 19 ng/kg bw/day and P95 values reaching up to 54 ng/kg bw/day. These elevated exposures were primarily associated with food categories such as hempseed, spices, and herbal infusions, as well as certain cereal-based snacks [37]. When severe TA contamination in spinach-based foods in Italy occurred, the calculated *EDI* for the Italian population exceeded the *ARfD* by factors ranging from 2 to more than 1200, with toddlers showing the highest mean and 95th percentile exposures [26]. Although such events present rare, accidental contamination linked to the presence of the weeds from the Solanaceae family, predominantly *D. stramonium*, they illustrate how high acute exposure can occur in food categories that are not routinely associated with TA contamination. A recent study from Chad analyzed millet, pearl millet, and maize products and found mean At and Sc levels of approximately 2400–2600 and 0.7–1.0 µg/kg, respectively. *EDI* reached 0.07 µg/kg bw/day in children and 0.132 µg/kg bw/day in adults, indicating a potential health concern for regular consumers [43].

In Serbia, a risk assessment study was conducted on 103 maize products and showed that the group *ARfD* was exceeded in 21.4% of exposure scenarios for children, and 11.7–17.5% for adolescents and adults, with the maximum intakes reaching 9- to 19-fold above the group *ARfD* [44]. Another study, dealing with corn puffs, reported quantifiable At in 22% of the samples, while scopolamine was detectable only below the LOQ, with full co-occurrence and an At:Sc ratio of 3.4–3.9, which is indicative of *D. stramonium* contamination. Even in the most contaminated sample (2.05 µg/kg total TAs), the estimated exposure reached only 32% of the *ARfD* for children and decreased to below 10% in adults [45].

Unlike the other cereal commodities, such as maize, millet, buckwheat, or composite gluten-free products, for which both the occurrence and exposure assessments have been reported, no comparable data exist for barley. Consequently, the calculated *EDI* values for barley-based foods and beverages have not previously been documented, and the results of this study present the first available estimates for this cereal. These findings, therefore, contribute new evidence to an area where information has so far been limited.

### 4.4. Statistical Analysis

Both the one-way and factorial ANOVA indicated that the *EDI* values varied significantly across the population groups, production systems, exposure scenarios, and calculated distribution metrics. These statistical differences reflect the expected influence of the consumption patterns, body weight, TA concentrations, and the structure of the exposure assumptions on the resulting *EDI* estimates. As anticipated, the organically produced barley samples consistently generated higher *EDI* values than the conventional ones, which is in line with the higher TA concentrations measured in the samples from the organic production. The variation among the population groups corresponds to the known differences in the intake and body mass, while the different exposure scenarios and calculated metrics produced the anticipated gradient from central tendency to increasingly conservative upper-bound estimates.

Factorial ANOVA further showed that the majority of the combined effects were statistically significant, although no interaction was observed between the population groups and production systems, nor between the exposure scenarios and production systems, indicating that these factors influenced *EDI* independently rather than synergistically.

Overall, the statistical patterns are consistent with the structure of the exposure model and serve to characterize the behavior and variability of the calculated *EDI* distributions, rather than implying differences in the actual exposure.

## 5. Conclusions

Although neither organic nor conventional barley samples exhibited markedly elevated concentrations of At and Sc, and the *EDI* values calculated under realistic and conservative consumption scenarios did not exceed the *ARfD*, these results should not be interpreted as evidence that barley inherently poses no toxicological relevance with respect to TAs. The currently available data on TA occurrence and exposure across cereal commodities remain limited, and the extent to which different grains may contribute to acute dietary intake is still not fully characterized.

In this context, one of the key challenges in risk characterization is the scarcity of barley-specific data from other regions, which limits the direct international comparison and comprehensive benchmarking of the exposure levels. Therefore, future multi-regional and multi-commodity monitoring would substantially improve the robustness of the TA risk assessment for cereals.

For this reason, maintaining systematic monitoring and control measures for barley, alongside other widely consumed cereals, remains essential to ensure consumer protection, particularly in terms of the potential variability in contamination arising from the agronomic conditions and accidental co-harvesting of Solanaceae weeds. Preventive risk management should primarily focus on minimizing the presence of the TA-producing weeds in barley fields through appropriate agronomic practices and careful harvesting, while post-harvest operations, such as cleaning, sorting, and dehulling, may further reduce the contamination in barley-based food products.

As no prior exposure assessment has been published for barley, the present study provides the first reference values for this cereal, while highlighting the need for broader, long-term, and regionally diverse investigations to more comprehensively characterize the potential for TA-related health risks.

## Figures and Tables

**Table 1:** Liquid chromatography electrospray ionization tandem mass spectrometric parameters for the analysis of At and Sc in MRM mode.

TA	Molecular Formula	Molecular Weight (g/mol)	Precursor Ion [M+H^+^] (*m*/*z*)	Product Ion (*m*/*z*)	Fragmentation Voltage (V)	Collision Energy (V)	Cell Acquisition (V)	Retention Time (min)
Atropine	C_17_H_23_NO_3_	289.37	290.20	77.00	135.00	68.00	5.00	5.30
				93.20		35.00		
				124.20		26.00		
Scopolamine	C_17_H_21_NO_4_	303.36	304.20	103.20	120.00	44.00	5.00	4.10
				138.20		24.00		
				156.00		15.00		

**Table 2:** Linearity and recovery.

TA	Linearity, (1–20 µg/kg) R^2^	Mean Recoveries, Rec ± %RSDr	Up, %
Atropine	0.9979	105.94 ± 9.77	40.80
Scopolamine	0.9996	100.14 ± 14.84	43.00

**Table 3:** The maximum and minimum values detected for At, Sc, and the sum of At and Sc in organic and conventional barley samples.

	Atropine (µg/kg)	Scopolamine (µg/kg)	Sum of Atropine and Scopolamine (µg/kg)	
Maximum levels	1.47 1.31	1.76 1.03	3.20 2.07	Organic production Conventional production
Minimum levels	0.58 0.62	0.60 0.43	1.18 1.07	Organic production Conventional production

**Table 4:** The calculated *EDI* values for the sum of At and Sc under three exposure scenarios and across seven population groups.

Population Group	Calculated Parameters	*EDI* RCS OP Day 1 and Day 2	*EDI* WCS OP Day 1 and Day 2	*EDI* EWCS OP Day 1 and Day 2	*EDI* RCS CP Day 1 and Day 2	*EDI* WCS CP Day 1 and Day 2	*EDI* EWCS CP Day 1 and Day 2
TNS	Maximum value	1.55 3.51	3.52 7.95	18.86 17.76	1.00 2.27	2.28 5.14	12.20 11.49
	Median value	0.00 0.06	0.00 0.11	0.00 3.91	0.00 0.04	0.00 0.07	0.00 2.53
	Average value	0.26 0.29	0.55 0.58	1.93 4.26	0.17 0.19	0.35 0.38	1.25 2.76
	P75	0.12 0.25	0.29 0.30	2.91 7.04	0.08 0.16	0.19 0.19	1.88 4.55
	P95	1.36 1.43	3.09 3.05	7.37 10.19	0.88 0.93	2.00 1.97	4.77 6.59
CNS	Maximum value	3.02 1.77	6.85 3.99	21.30 17.45	1.95 1.14	4.43 2.58	13.78 11.29
	Median value	0.00 0.03	0.01 0.06	0.65 1.52	0.00 0.02	0.01 0.04	0.42 0.98
	Average value	0.17 0.23	0.37 0.50	2.09 2.37	0.11 0.15	0.24 0.32	1.35 1.53
	P75	0.06 0.09	0.09 0.24	3.00 3.63	0.04 0.06	0.06 0.15	1.94 2.35
	P95	1.20 1.34	2.65 3.03	7.82 8.36	0.78 0.86	1.72 1.96	5.06 5.40
ADNS	Maximum value	7.71 6.10	10.60 8.38	96.39 76.19	4.99 3.94	6.86 5.42	62.35 49.29
	Median value	0.10 0.30	0.17 0.46	1.58 2.61	0.07 0.20	0.11 0.30	1.02 1.69
	Average value	0.57 0.61	0.86 0.88	6.76 7.57	0.37 0.39	0.56 0.57	4.38 4.90
	P75	0.79 0.86	1.17 1.32	9.01 10.67	0.51 0.55	0.76 0.85	5.83 6.90
	P95	2.37 2.34	3.32 3.22	29.97 29.24	1.53 1.51	2.15 2.08	19.39 18.92
ANS	Maximum value	4.53 2.42	4.94 5.30	30.19 30.19	2.93 1.56	3.19 3.43	19.53 19.53
	Median value	0.02 0.01	0.05 0.02	1.45 0.63	0.02 0.01	0.03 0.01	0.94 0.41
	Average value	0.25 0.25	0.48 0.50	2.40 1.92	0.16 0.16	0.31 0.32	1.56 1.24
	P75	0.24 0.16	0.44 0.26	2.80 2.25	0.15 0.11	0.28 0.17	1.81 1.46
	P95	1.09 1.62	2.48 2.87	10.35 5.18	0.71 1.05	1.60 1.86	6.69 3.35
ENS	Maximum value	3.89 3.87	5.18 4.24	47.06 28.78	2.52 2.50	3.35 2.74	30.44 18.62
	Median value	0.10 0.32	0.23 0.48	1.49 1.66	0.06 0.20	0.15 0.31	0.97 1.08
	Average value	0.44 0.50	0.68 0.74	4.74 5.40	0.28 0.32	0.44 0.48	3.06 3.49
	P75	0.71 0.77	1.06 1.15	7.64 9.13	0.46 0.50	0.69 0.75	4.94 5.90
	P95	1.68 1.71	2.42 2.40	20.54 21.33	1.09 1.11	1.57 1.55	13.29 13.80
VNS	Maximum value	9.14 9.14	9.97 9.97	89.05 36.47	5.91 5.91	6.45 6.45	57.60 23.59
	Median value	0.19 0.02	0.23 0.05	1.89 1.25	0.12 0.01	0.15 0.03	1.22 0.81
	Average value	0.62 0.45	0.90 0.64	6.54 4.43	0.40 0.29	0.58 0.41	4.23 2.86
	P75	0.74 0.49	1.16 1.08	8.63 5.65	0.48 0.32	0.75 0.70	5.58 3.66
	P95	2.47 1.89	3.40 2.64	22.90 18.53	1.60 1.22	2.20 1.71	14.82 11.99
PNS	Maximum value	1.05 1.18	2.38 2.67	9.57 7.68	0.68 0.76	1.54 1.73	6.19 4.97
	Median value	0.02 0.01	0.05 0.03	1.06 0.65	0.01 0.01	0.03 0.02	0.69 0.42
	Average value	0.16 0.20	0.34 0.43	1.37 1.47	0.10 0.13	0.22 0.28	0.88 0.95
	P75	0.21 0.29	0.35 0.53	1.69 2.14	0.14 0.19	0.23 0.34	1.09 1.38
	P95	0.70 1.11	1.45 2.48	5.69 6.94	0.45 0.72	0.94 1.60	3.68 4.49

*EDI*—Estimated daily intake (ng/kg bw/day); RCS OP/CP—Realistic-Case Scenario for organic/conventional production; WCS OP/CP—Worst-Case Scenario for organic/conventional production; EWCS OP/CP—Extremely Worst-Case Scenario for organic/conventional production; TNS—toddlers (1 to <3 y); CNS—children (3–9 y); ADNS—adolescents (10–17 y); ANS—adults (18–64 y); ENS—elderly (65–74 y); VNS—vegetarians; PNS—pregnant women; P75—75th percentile; P95—95th percentile.

**Table 5:** Spearman’s correlation analysis.

Variable	Moisture (%)	Protein Dry Basis (%)	Dry Gluten As Is (%)	Hardness -	Starch Dry Basis (%)	Wet Gluten As Is (%)	W Fixed = 14 -	Zeleny Fixed = 14 mL	NDF Dry Basis (%)	Sum of At and Sc
Moisture (%)	1.00	−0.48	−0.14	**−0.86**	0.62	−0.14	0.19	**0.76**	−0.14	−0.10
Protein Dry basis (%)	−0.48	1.00	**0.74**	0.33	**−0.74**	0.52	−0.02	−0.50	0.50	**0.76**
Dry gluten As is (%)	−0.14	**0.74**	1.00	0.07	−0.57	**0.90**	0.00	−0.10	**0.81**	0.62
Hardness -	**−0.86**	0.33	0.07	1.00	−0.36	0.24	−0.31	−0.50	−0.17	0.10
Starch Dry basis (%)	0.62	**−0.74**	−0.57	−0.36	1.00	−0.38	0.29	0.33	−0.67	−0.48
Wet gluten As is (%)	−0.14	0.52	**0.90**	0.24	−0.38	1.00	−0.12	0.05	0.62	0.40
W Fixed = 14 -	0.19	−0.02	0.00	−0.31	0.29	−0.12	1.00	−0.36	0.00	0.33
Zeleny Fixed = 14 mL	**0.76**	−0.50	−0.10	−0.50	0.33	0.05	−0.36	1.00	−0.10	−0.21
NDF Dry basis (%)	−0.14	0.50	**0.81**	−0.17	−0.67	0.62	0.00	−0.10	1.00	0.33
Sum of At and Sc	−0.10	**0.76**	0.62	0.10	−0.48	0.40	0.33	−0.21	0.33	1.00

The values shown in bold indicate the statistically significant correlations at *p* < 0.05. W—alveograph energy value; NDF—neutral detergent fiber; At—atropine; Sc—scopolamine.

## Data Availability

The original contributions presented in this study are included in the article. Further inquiries can be directed to the corresponding authors.

## References

[1] FAO (2022). Agricultural Production Statistics 2000–2021; FAOSTAT Analytical Briefs.

[2] FAO (2024). Agricultural Production Statistics 2010–2023; FAOSTAT Analytical Briefs.

[3] Bernád V., Al-Tamimi N., Langan P., Gillespie G., Dempsey T., Henchy J., Harty M., Ramsay L., Houston K., Macaulay M. (2024). Unlocking the genetic diversity and population structure of the newly introduced two-row spring European HerItage Barley collecTion (ExHIBiT). Front. Plant Sci..

[4] Lukinac J., Jukić M. (2022). Barley in the production of cereal-based products. Plants.

[5] Pacanoski Z. (2017). Introductory chapter: Actual issues (moments) in herbicide resistance weeds and crops. Herbicide Resistance in Weeds and Crops.

[6] Casado N., Gañán J., Morante-Zarcero S., Sierra I. (2020). New advanced materials and sorbent-based microextraction techniques as strategies in sample preparation to improve the determination of natural toxins in food samples. Molecules.

[7] Martinello M., Borin A., Stella R., Bovo D., Biancotoo G., Gallina A., Mutinelli F. (2017). Development and validation of a QuEChERS method coupled to liquid chromatography and high resolution mass spectrometry to determine pyrrolizidine and tropane alkaloids in honey. Food Chem..

[8] Chavez B.G., Dias S.L., D’Auria J.C. (2024). The evolution of tropane alkaloids: Coca does it differently. Curr. Opin. Plant Biol..

[9] Kim N., Estrada O., Chavez B., Stewart C., D’Auria J.C. (2016). Tropane and granatane alkaloid biosynthesis: A systematic analysis. Molecules.

[10] Shim K.H., Kang M.J., Sharma N., An S.S.A. (2022). Beauty of the beast: Anticholinergic tropane alkaloids in therapeutics. Nat. Prod. Bioprospect..

[11] Kohnen-Johannsen K.L., Kayser O. (2019). Tropane alkaloids: Chemistry, pharmacology, biosynthesis and production. Molecules.

[12] González-Gómez L., Morante-Zarcero S., Pérez-Quintanilla D., Sierra I. (2022). Occurrence and chemistry of tropane alkaloids in foods, with a focus on sample analysis methods: A review on recent trends and technological advances. Foods.

[13] de Nijs M., Crews C., Dorgelo F., MacDonald S., Mulder P.P.J. (2023). Emerging issues on tropane alkaloid contamination of food in Europe. Toxins.

[14] Cirlini M., Demuth T.M., Biancardi A., Rychlik M., Dall`Asta C., Bruni R. (2018). Are tropane alkaloids present in organic foods? Detection of scopolamine and atropine in organic buckwheat (*Fagopyron esculentum* L.) products by UHPLC-MS/MS. Food Chem..

[15] Lamp J., Knappstein K., Walte H.G., Krause T., Steinberg P., Schwake-Anduschus C. (2021). Transfer of tropane alkaloids (atropine and scopolamine) into the milk of subclinically exposed dairy cows. Food Control..

[16] Selmar D., Radwan A., Hijazin T., Abouzeid S., Yahyazadeh M., Lewerenz L., Kleinwächter M., Nowak M. (2019). Horizontal natural product transfer: Intriguing insights into a newly discovered phenomenon. J. Agric. Food Chem..

[17] Lewerenz L., Abouzeid S., Yahyazadeh M., Hijazin T., Selmar D. (2022). Novel cognitions in allelopathy: Implications from the “Horizontal Natural Product Transfer”. Plants.

[18] Jandrić Z., Rathor M.N., Chhem-Kieth S., Adu-Gyamfi J., Mayr L., Resch C., Bado S., Švarc-Gajić J., Cannavan A. (2013). Uptake of ^14^C-atropine and/or its transformation products from soil by wheat (*Triticum aestivum* var Kronjet) and their translocation to shoots. J. Environ. Sci. Health Part B.

[19] Yahyazadeh M., Nowak M., Kima H., Selmar D. (2017). Horizontal natural product transfer: A potential source of alkaloidal contaminants in phytopharmaceuticals. Phytomedicine.

[20] Selmar D., Wittke C., Beck-von Wolffersdorff I., Klier B., Lewerenz L., Kleinwächter M., Nowak M. (2019). Transfer of pyrrolizidine alkaloids between living plants: A disregarded source of contaminations. Environ. Pollut..

[21] Hardie A.G., Olifant K., Smith J.F.N., Hoffman J.E. (2023). Scientific evidence of soil transfer of pyrrolizidine alkaloids originating from weed species to rooibos tea. S. Afr. J. Bot..

[22] Hama J.R., Strobel B.W. (2021). Occurrence of pyrrolizidine alkaloids in ragwort plants, soils and surface waters at the field scale in grassland. Sci. Total Environ..

[23] Fernández-Pintor B., Morante Zarcero S., Sierra I. (2025). Tropane and pyrrolizidine alkaloids in edible flowers and flower-derived foods: A food safety perspective. Foods.

[24] Mulder P.P.J., de Nijs M., Castellari M., Hortos M., MacDonald S., Crews C., Hajslova J., Stranska M. (2016). Occurrence of tropane alkaloids in food. EFSA Support. Publ..

[25] Abia W.A., Montgomery H., Nugent A.P., Elliott C.T. (2021). Tropane alkaloids contamination of agricultural commodities and food products in relation to consumer health: Learnings from the 2019 Uganda food aid outbreak. Compr. Rev. Food Sci. Food Saf..

[26] Caprai E., Prizio I., Peloso M., Sonfack G.M., Bonan S., Benini N., Ghidini S., Varrà M.O., Zanardi E., Lanza G.T. (2024). Case reports of tropane alkaloid contamination in spinach from Italy and its potential implications for consumer health. Food Control..

[27] Vivares S., Jousset N., Abbara C., Renard L., Malbanque S., Ferec S., Briet M., Drevin G. (2025). Accidental foodborne poisoning by atropine and scopolamine: A fatal case report. J. Forensic Leg. Med..

[28] Rapid Alert System for Food and Feed (RASFF). https://food.ec.europa.eu/food-safety/rasff_en.

[29] Kowalczyk E., Kwiatek K. (2022). Scopolamine and atropine in feeds–determination with liquid chromatography mass spectrometry. Food Addit. Contam. Part A.

[30] Vuković G., Stojanović T., Konstantinović B., Bursić V., Puvača N., Popov M., Samardžić N., Petrović A., Marinković D., Roljević Nikolić S. (2022). Atropine and scopolamine in maize products from the retail stores in the Republic of Serbia. Toxins.

[31] (2023). Commission Implementing Regulation (EU) 2023/2783 Laying Down the Methods of Sampling and Analysis for the Control of the Levels of Plant Toxins in Food and Repealing Regulation (EU) 2015/705. OJEU. https://eur-lex.europa.eu/eli/reg_impl/2023/2783/oj.

[32] Vuković G., Bursić V., Stojanović T., Petrović A., Gvozdenac S., Starović M., Kuzmanović S., Aleksić G. (2018). LC-MS/MS determination of tropane alkaloids in maize crop. Contemp. Agric..

[33] SANTE 11312/2021 v2. Analytical Quality Control and Method Validation Procedures for Pesticide Residues Analysis in Food and Feed. https://www.eurl-pesticides.eu/docs/public/tmplt_article.asp?CntID=727.

[34] Foddy H., Adams S., Trivedi E., Hancock P. (2023). Determination of Pesticide Residues in Wheat Flour and Cucumber After Extraction with QuEChERS and Clean-Up with Oasis™ PRiME HLB SPE.

[35] Zeković M., Gurinović M., Milešević J., Glibetić M. (2021). National Food Consumption Survey among children from 1 to 9 years old in Serbia. EFSA Support. Publ..

[36] Zeković M., Gurinović M., Milešević J., Kadvan A., Glibetić M. (2022). National Food Consumption Survey among 10–74 years old individuals in Serbia. EFSA Support. Publ..

[37] Arcella D., Altieri A., Horváth Z., EFSA (2018). Human acute exposure assessment to tropane alkaloids. EFSA J..

[38] EFSA CONTAM Panel (2013). Scientific opinion on tropane alkaloids in food and feed. EFSA J..

[39] FAO, WHO (2020). Joint FAO/WHO Expert meeting on tropane alkaloids. Food Saf. Qual. Ser..

[40] Han S., Jang S., Oh S., Lee J., Lee H.J., Koo Y.E., Kim B.H. (2024). Occurrence and health risk assessment of tropane alkaloids in cereal foods consumed in Korea. Food Chem. Toxicol..

[41] Stratton J., Clough J., Leon I., Sehlanova M., MacDonald S. (2017). Monitoring of Tropane Alkaloids in Foods.

[42] Vera-Baquero F.L., Pérez-Quintanilla D., Morante-Zarcero S., Sierra I. (2025). Assessment of atropine and scopolamine in commercial multigrain cereal-based baby products using UHPLC-TQ-MS/MS and solid phase extraction with MCM-41 mesostructured silica as sorbent. Food Chem..

[43] Ambadi W.I., Nfombouot H.P.N., Djomptchouang H.T., Ali K.H., Abia W.A. (2025). Occurrence and health risk assessment of tropane alkaloids (atropine and scopolamine) in cereal-based foods consumed by children and adults in N’djamena, Chad. Ann. Food Process. Preserv..

[44] Torović L., Bursić V., Vuković G. (2023). Health concerns associated to tropane alkaloids in maize food products in Serbia. Heliyon.

[45] Stojanović T., Vuković G., Petrović A., Konstantinović B., Puvača N., Marinković D., Gvozdenac S., Bursić V. (2021). Determination of tropane alkaloids in corn puffs by the LC-MS/MS. Matica Srp. J. Nat. Sci..

